# Effectiveness of mental health first aid training for nursing students in northwestern China: a quasi-experimental study

**DOI:** 10.3389/fmed.2026.1907730

**Published:** 2026-08-24

**Authors:** Shadiyemu Tuerxun, Xi Chen, Mingzhe Wu, Haijing Ma, Yujia Chen, Ping Song, Yunxia Li, Yim-Wah Mak

**Affiliations:** 1 School of Nursing, Lanzhou University, Lanzhou, China; 2 School of Nursing, The Hong Kong Polytechnic University, Kowloon, Hong Kong SAR, China; 3 Department of Interventional Medicine, The First Hospital of Lanzhou University, Lanzhou, China

**Keywords:** mental health first aid (MHFA), mental health literacy, nursing students, quasi-experimental study, self-efficacy

## Abstract

**Background:**

Mental health first aid (MHFA) is an internationally recognized training program that equips participants with skills to recognize and respond to individuals experiencing mental health problems. However, evidence on the effectiveness of MHFA training among nursing students in mainland China remains limited.

**Objectives:**

To evaluate the longitudinal effects of MHFA training on mental health literacy, self-stigma of seeking help, psychological well-being, and self-efficacy among nursing students.

**Design:**

A single-group quasi-experimental pretest-posttest study.

**Setting:**

A school of nursing at a university in northwestern China.

**Participants:**

Forty-nine nursing students were recruited and provided informed consent.

**Methods:**

Participants received a 3-day face-to-face MHFA training program. Outcomes were assessed at baseline, immediately post-intervention, 1-month follow-up, and 3-month follow-up using validated scales. Linear mixed models were used to examine longitudinal changes, with time as a fixed effect and participant as a random intercept.

**Results:**

Self-efficacy significantly improved from baseline to post-intervention (*p* = 0.006). Mental health literacy showed a non-significant increase post-intervention and significantly declined from post-intervention to the 3-month follow-up (*p* = 0.006). MHFA knowledge scores decreased continuously during the follow-up period, with a significant difference between post-intervention and the 3-month follow-up (*p* = 0.019). No significant changes were observed in self-stigma of seeking help or psychological well-being across time points.

**Conclusion:**

The 3-day MHFA training was associated with a significant increase in nursing students’ self-efficacy, but its impact on mental health literacy, self-stigma, and psychological well-being was limited. A modest but statistically significant decline in knowledge scores was observed during the follow-up period, suggesting the need for regular reinforcement. Integrating a localized MHFA program into nursing curricula may help strengthen students’ confidence in providing mental health support.

## Highlights

- Face-to-face MHFA training was associated with a significant increase in nursing students’ self-efficacy.
- Self-efficacy scores remained numerically above baseline throughout the 3-month follow-up.
- Mental health literacy showed a non-significant increase after the intervention.
- MHFA knowledge scores showed a modest but statistically significant decline during the follow-up period.
- Linear mixed models effectively handled missing data in this longitudinal design.

## 1 Introduction

According to the latest data from the Global Burden of Disease Study 2023, mental disorders are the leading cause of years lived with disability (YLDs) worldwide, accounting for 17.3% of all-cause YLDs (1). In China, the lifetime prevalence of mental disorders among adults aged 18 years and above is 16.6% (2), and mental disorders account for 7 of the top 20 causes of disability-adjusted life years (DALYs), a measure of total healthy life years lost from onset to death (2). Mental disorders are characterized by high rates of disability, relapse, and suicide. However, the majority of individuals with mental illness lack a clear understanding of early signs and symptoms, have low mental health literacy (MHL), or hold strong stigma toward mental illness (3). As a result, only a small proportion seek medical or professional help at the onset of symptoms, missing the optimal window for treatment. This situation may result in adverse consequences such as death, suicide, or suspension of schooling due to psychological distress. Nursing students, as a core group within the future healthcare workforce, play a key role in determining whether patients with mental disorders receive appropriate care, and their attitudes are a critical influencing factor. However, research has shown that nursing students, like the general public, hold misconceptions about individuals with mental illness, often harbor stigmatizing attitudes, and exhibit low levels of empathy. When interacting with patients, they tend to display avoidance and anxiety, which not only compromises the quality of care they provide but may also negatively influence their future career choices (4). Furthermore, the demanding and high-pressure nature of nursing education places this group at a higher risk of developing mental health problems than the general population, while the associated stigma and concerns about professional repercussions further deter them from seeking professional psychological help (5). A systematic review showed that direct contact with patients with mental disorders combined with mental health education and practical training, including case study discussions, role playing, and video based instruction, can more effectively reduce the stigma and stigmatizing attitudes of nursing students toward people with mental disorders (6).

Mental health first aid (MHFA) is an internationally recognized training program designed to equip the public with the skills to recognize, understand, and respond to individuals experiencing mental health problems or crises, and to provide initial support before professional help is available (7). The program integrates evidence-based MHFA guidelines developed through Delphi expert consensus with interactive teaching methods such as role playing and group discussions, and has been implemented in at least 25 countries and regions worldwide, including Australia, the United Kingdom, the United States, Canada, and several Asian countries (8, 9). Research has demonstrated that the MHFA program is efficacious in improving participants’ ability to recognize mental health problems and their mental health literacy, increasing their confidence in addressing mental health issues, and reducing stigma toward individuals with mental illness and their peers (7, 10, 11).

Previous studies have applied the MHFA program to medical and nursing students, yielding positive effects in improving mental health literacy, enhancing willingness and confidence to provide help, and reducing stigma (5, 8). However, the majority of this research has been conducted in Western countries, and evidence evaluating the effectiveness of MHFA training specifically among nursing students in mainland China remains limited. Furthermore, most existing studies have only examined short-term changes pre- and post-intervention, and there is a lack of longitudinal follow-up data to reflect the durability of training effects. To this end, the present study adopted a single-group quasi-experimental pretest-posttest design and recruited nursing students at a comprehensive university in northwestern China. The longitudinal effects of MHFA training on mental health literacy, self-stigma of seeking help, psychological well-being, and self-efficacy were assessed at four time points: baseline, immediately post-intervention, 1 month post-intervention, and 3 months post-intervention. The study aimed to enhance nursing students’ professional confidence in responding to mental health problems, enabling them to take appropriate measures when facing psychological distress themselves, and to provide empirical evidence for the localized design of MHFA training programs within nursing education in mainland China.

## 2 Methods

### 2.1 Design

This study employed a single-group quasi-experimental pretest-posttest design. All participants received the same MHFA training intervention and completed assessments at four time points: baseline (T0), immediately post-intervention (T1), 1 month post-intervention (T2), and 3 months post-intervention (T3). The study was conducted between July and October 2025.

### 2.2 Participants

This study recruited full-time students from the nursing school of a comprehensive university in northwestern China. Recruitment information was disseminated through class announcements, and a convenience sampling method was used to enroll students who met the inclusion criteria and volunteered to participate. Eligible participants were those who: (a) were at least 18 years of age; (b) were full-time registered nursing students; and (c) provided informed consent and voluntarily agreed to participate in the study. G*Power 3.1 software was used to calculate the required sample size. With an effect size *f* = 0.25 (medium effect), a statistical power (1 − β) of 0.80, an *α* level of 0.05, and 4 repeated measurements, the minimum required sample size was determined to be 24 participants. Considering an anticipated attrition rate of 20–30% in this longitudinal study, the recruitment target was set at a minimum of 35 participants. Ultimately, a total of 49 nursing students voluntarily enrolled and provided informed consent to participate in this study.

### 2.3 Intervention

The intervention in this study was a face-to-face MHFA training program, with content adapted from the Hong Kong version. The training was delivered in English by Dr. Yim-Wah MAK, a certified MHFA instructor, with research team members Xi CHEN and Yunxia LI providing real-time translation and interpretation to facilitate communication between the instructor and participants. Printed training materials were distributed to each student in their original version prior to the training. The training adopted an interactive small-group teaching format and was conducted over three consecutive days (July 8–10, 2025) at a designated teaching building within the nursing school, totaling approximately 19 h. All sessions were delivered by the same team of instructors to ensure consistency of the training content. The training covered the identification of mental health problems, the application of the MHFA action plan, and the management of common psychological crises. The detailed course schedule is presented in Table 1.

**Table 1:** Schedule of the MHFA training program.

Date	Content	Facilitator
July 8	Mental Health Problems & MHFA; Depression	Dr. Yim-Wah MAK
	MHFA for Depression: Applying the ALGEE Action Plan	
July 9	MHFA: Crisis Associated with Depression (Applying ALGEE)	
	MHFA: Introduction to Anxiety Problems	
July 10	MHFA for Anxiety Problems and Associated Crises; Substance Abuse and Its MHFA; Psychosis and Associated Crises	
	Quiz and Course Evaluation	

### 2.4 Procedures

In June 2025, the researchers disseminated recruitment information through class group notifications. Students interested in participating completed the registration via a QR code and signed an electronic informed consent form and confidentiality agreement. Before the training formally began, participants completed the baseline electronic questionnaire (T0). Following the baseline assessment, participants attended a 3-day face-to-face MHFA training program. The training consisted of six sessions, each lasting approximately 3 h, conducted in the morning and afternoon by the same team of instructors. Upon completion of the 3-day training, participants immediately completed the second questionnaire assessment (T1). The researchers then sent follow-up questionnaire links via the Wenjuanxing platform at 1 month (T2) and 3 months (T3) post-training. During each follow-up period, one to two reminders were sent through a WeChat group to minimize attrition. Questionnaires at all time points were self-administered by the participants.

All 49 enrolled participants attended the 3-day training and completed the program. Attendance was recorded at each session, and full attendance was achieved across all sessions. The training was delivered by the same certified instructor following a standardized curriculum based on the Hong Kong version of the MHFA program. To ensure consistency of delivery, all sessions followed the pre-specified course schedule presented in Table 1. Participants were not exposed to any additional formal mental health teaching or training during the 3-month follow-up period, as confirmed by the follow-up questionnaire, which included an item asking whether participants had attended any mental health-related training outside the study.

### 2.5 Variables and data collection instrument

All instruments used in this study were administered in Chinese. The Mental Health Literacy Scale, the Self-Stigma of Seeking Help Scale, and the Scales of Psychological Well-being were adopted from previously validated Chinese versions, as detailed in the respective sections below. The self-efficacy scale was originally developed in Chinese as part of the 5A Mental Health Ambassador Program at the Hong Kong Polytechnic University and was used in its original language version. The MHFA knowledge test was developed in Chinese by the research team specifically for this study.

#### 2.5.1 Baseline variables

Baseline data on participants’ sociodemographic characteristics and mental health-related background information were collected, including age, sex, grade, ethnicity, religious belief, annual household income per capita, history of mental health disorders, contact experience with individuals with mental disorders, use of mental health services, awareness of MHFA, and prior learning and training experiences related to mental health courses.

#### 2.5.2 Primary variable

Mental health literacy was assessed using the Chinese version of the Mental Health Literacy Scale (MHLS-C), which was translated and validated by Wang et al. (12) based on the original scale developed by O’Connor and Casey (13). The MHLS-C consists of 29 items, comprising two subscales: core literacy (22 items) and social acceptance (7 items), and covers four dimensions—knowledge of mental disorders (13 items), ability to seek information and help (4 items), recognition of mental disorders (5 items), and acceptance of individuals with mental illness (7 items). The scale adopts a mixed scoring method, with the first 13 items rated on a 4-point Likert scale and the remaining 16 items on a 5-point Likert scale, with some items reverse-scored. The total score ranges from 29 to 132, with higher scores indicating a higher level of mental health literacy. The Cronbach’s alpha for the total scale in this study was 0.808.

#### 2.5.3 Secondary variable

Self-stigma of seeking professional psychological help was measured using the Self-Stigma of Seeking Help Scale (SSOSH), developed by Vogel et al. (14). The SSOSH is a unidimensional scale consisting of 10 items, each rated on a 5-point Likert scale ranging from 1 (strongly disagree) to 5 (strongly agree). The total score ranges from 10 to 50, with higher scores indicating a greater degree of self-stigma associated with seeking professional psychological help. The Cronbach’s alpha for this scale in the present study was 0.91.

Psychological well-being was measured using the 18-item short version of the Scales of Psychological Well-being (SPWB), originally developed by Ryff and Keyes (15). The scale comprises six dimensions, namely autonomy, environmental mastery, personal growth, positive relations with others, purpose in life, and self-acceptance, each consisting of three items. Items are rated on a 7-point Likert scale ranging from 1 (strongly agree) to 7 (strongly disagree). The total score ranges from 18 to 126, with higher scores indicating greater psychological well-being. The Cronbach’s alpha for this scale in the present study was 0.945.

Self-efficacy in providing mental health support to peers was measured using the “Self-efficacy in delivering the 5As and promoting the 5Ways toward wellbeing to their peers” scale, which was adopted from the 5A Mental Health Ambassador Program at the Hong Kong Polytechnic University. The scale consists of seven items, each rated on a 6-point Likert scale ranging from 1 (never) to 6 (all the time). The total score ranges from 7 to 42, with higher scores indicating greater self-efficacy in delivering mental health support to peers. The Cronbach’s alpha for this scale in the present study was 0.941.

MHFA knowledge was assessed using a self-developed knowledge test designed by the research team based on the content of the MHFA training program. The test consists of 20 single-choice questions covering core training topics such as basic concepts of MHFA, identification of common mental disorders, and crisis intervention strategies. Each correct answer was scored 5 points and each incorrect answer 0 points, yielding a total score ranging from 0 to 100, with higher scores indicating better MHFA knowledge. To reduce participant burden at baseline, when multiple validated scales were already being administered, the knowledge test was administered only at three time points: immediately post-intervention, 1 month post-intervention, and 3 months post-intervention.

### 2.6 Data analysis

Data were analyzed using SPSS 27.0. Categorical data were presented as frequencies and percentages. Linear mixed models were used to examine longitudinal changes in each outcome variable across the four time points. This approach utilizes all available participant data under the missing-at-random assumption, thereby avoiding the need to delete cases with missing values or perform imputation. In the model, time (T0, T1, T2, T3) was specified as a fixed effect, and participant ID was included as a random intercept. An unstructured covariance matrix was employed to account for the correlations among repeated measurements within each participant, and the restricted maximum likelihood method was used for parameter estimation. When a significant main effect of time was identified, *post hoc* pairwise comparisons were conducted using the Bonferroni correction. The significance level was set at *α* = 0.05.

All models converged successfully. An unstructured covariance matrix was selected to avoid imposing restrictive assumptions on the variance–covariance structure of the repeated measures, given that the four time points were unequally spaced and the correlations between adjacent measurements may differ from those between more distant time points.

### 2.7 Ethical compliance

This study was approved by the Ethics Review Committee of the School of Nursing, Lanzhou University (approval number: LZUHLXY20250215). Prior to the commencement of the study, the researchers disseminated recruitment information to postgraduate nursing students and distributed a study information sheet to those who expressed interest. The information sheet clearly outlined the study objectives, procedures, expected benefits, potential risks, and the voluntary nature of participation. Participants were informed that they could withdraw from the study unconditionally at any stage without any adverse consequences to their academic performance or personal interests. Written informed consent was obtained from all participants prior to enrollment. Throughout the study, all personal information and questionnaire data were anonymized and identified solely by code numbers, and the data were used exclusively for the purposes of this study and kept strictly confidential.

## 3 Results

### 3.1 Participant characteristics

**Figure 1:**
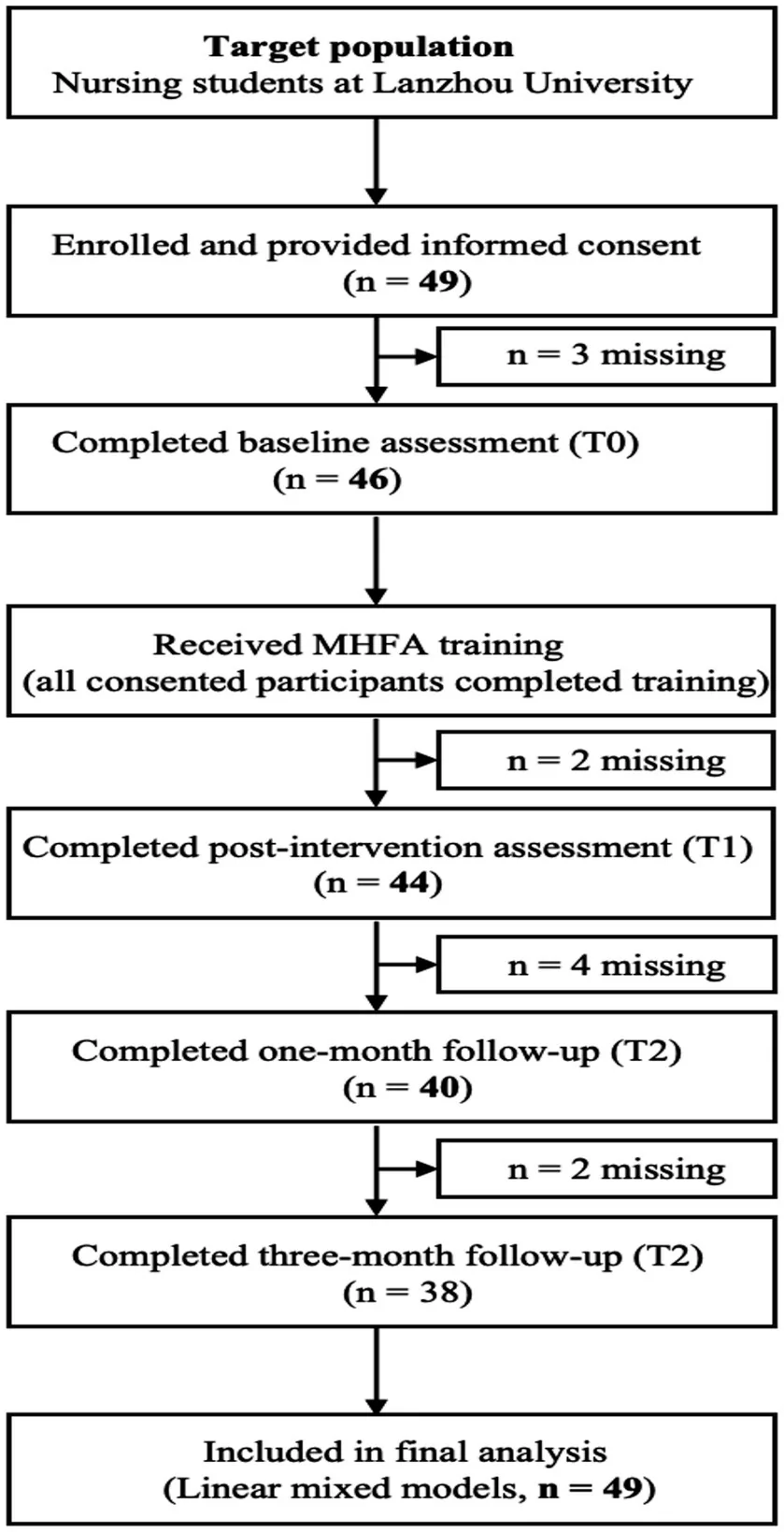
Participant flow diagram.

A total of 49 nursing students enrolled and provided informed consent. The number of participants completing assessments at each time point is shown in Figure 1. Of these, 46 completed the baseline (T0) assessment, 44 completed the post-intervention (T1) assessment, 40 completed the 1-month follow-up (T2), and 38 completed the 3-month follow-up (T3). The missing data pattern was non-monotone, meaning some participants who missed an assessment returned at a later time point. All enrolled participants completed the training, and 35 completed all four assessments, constituting the complete dataset. Baseline demographic characteristics are presented in Table 2.

**Table 2:** Baseline characteristics of participants (*N* = 49).

Variable	*n* (%)
Grade level	
First-year postgraduate	27 (55.10)
Second-year postgraduate	14 (28.57)
Third-year postgraduate	4 (8.16)
Third-year undergraduate	4 (8.16)
Age (years)	
21–25	43 (87.76)
≥26	6 (12.24)
Sex	
Male	5 (10.20)
Female	44 (89.80)
Ethnicity	
Han	45 (91.84)
Ethnic minorities	4 (8.16)
Annual household income per capita^a^	
<10,000 CNY	13 (26.53)
10,000–50,000 CNY	17 (34.69)
50,000–100,000 CNY	13 (26.53)
>100,000 CNY	6 (12.24)
History of mental health disorders	
No	47 (95.92)
Yes	2 (4.08)
Previous contact with individuals with mental disorders	
No	18 (36.73)
Yes	31 (63.27)
Use of mental health services	
Never used	35 (71.43)
Assisted others in using	12 (24.49)
Used personally	2 (4.08)
Awareness of MHFA	
Heard of	12 (24.49)
Never heard of	37 (75.51)
Encountered situations requiring MHFA	
No	39 (79.59)
Yes	10 (20.41)
Willingness to provide help in MHFA situations	
Uncertain	16 (32.65)
Willing	33 (67.35)
Completion of university-offered mental health courses	
Yes	28 (57.14)
No	21 (42.86)
Participation in mental health training outside curriculum	
No	49 (100.00)

MHFA, Mental health first aid. Percentages may not sum to 100% due to rounding. All participants selected “No” for participation in mental health training outside the curriculum. ^a^The income categories represent annual per capita household income. The lower income brackets reflect the regional economic context of northwestern China.

Participants ranged in age from 21 to 35 years (mean: 24.04 ± 2.96 years); 5 (10.20%) were male and 44 (89.80%) were female. The majority were first-year postgraduates (*n* = 27, 55.10%), followed by second-year postgraduates (*n* = 14, 28.57%), third-year postgraduates (*n* = 4, 8.16%), and third-year undergraduates (*n* = 4, 8.16%). Most were of Han ethnicity (*n* = 45, 91.84%), with 4 (8.16%) from ethnic minorities. The most frequently reported annual household income category was 10,000–50,000 CNY (*n* = 17, 34.69%), followed by less than 10,000 CNY and 50,000–100,000 CNY (both *n* = 13, 26.53%), and above 100,000 CNY (*n* = 6, 12.24%).

Regarding mental health-related background, 2 participants (4.08%) reported a history of mental health disorders; 31 (63.27%) had previous contact with individuals with mental disorders; 12 (24.49%) had assisted others in using mental health services, while 2 (4.08%) had used such services themselves. Twelve participants (24.49%) had heard of MHFA, and 10 (20.41%) had previously encountered situations requiring MHFA. When facing such situations, 33 (67.35%) expressed willingness to help, while 16 (32.65%) were uncertain. Twenty-eight participants (57.14%) had completed university-offered mental health courses, mainly Psychiatric Nursing. No participant reported prior mental health-related training outside the university curriculum.

### 3.2 Missing data patterns and baseline equivalence

A total of 49 participants were included in this study. Of these, 35 (71.4%) completed all four assessments, and 14 (28.6%) had missing data at one or more time points. Because questionnaire completion was voluntary, some participants did not respond at every time point despite reminders sent through the WeChat group. To evaluate the missing data mechanism, participants were categorized into a complete data group (*n* = 35) and a partial missing data group (*n* = 14), and the two groups were compared on baseline demographic characteristics and outcome scores.

Independent-samples t-tests were used for continuous variables, and chi-square tests or Fisher’s exact tests were used for categorical variables. Results showed that the partial missing data group reported significantly higher baseline self-stigma of seeking help than the complete data group (31.09 ± 10.21 vs. 22.26 ± 6.91, *t* = −2.685, *p* = 0.019). No statistically significant differences were found between the two groups in age, baseline mental health literacy, psychological well-being, self-efficacy, or any other baseline characteristics including sex, grade, ethnicity, and annual household income per capita (all *p* > 0.05).

These findings indicate that data missingness was associated with the observed high baseline level of self-stigma, suggesting that the data were not missing completely at random (MCAR). While this pattern is consistent with the missing-at-random (MAR) assumption, it cannot be confirmed solely on the basis of observed baseline characteristics, and missing-not-at-random (MNAR) mechanisms cannot be ruled out.

### 3.3 Longitudinal changes in outcome variables

**Figure 2:**
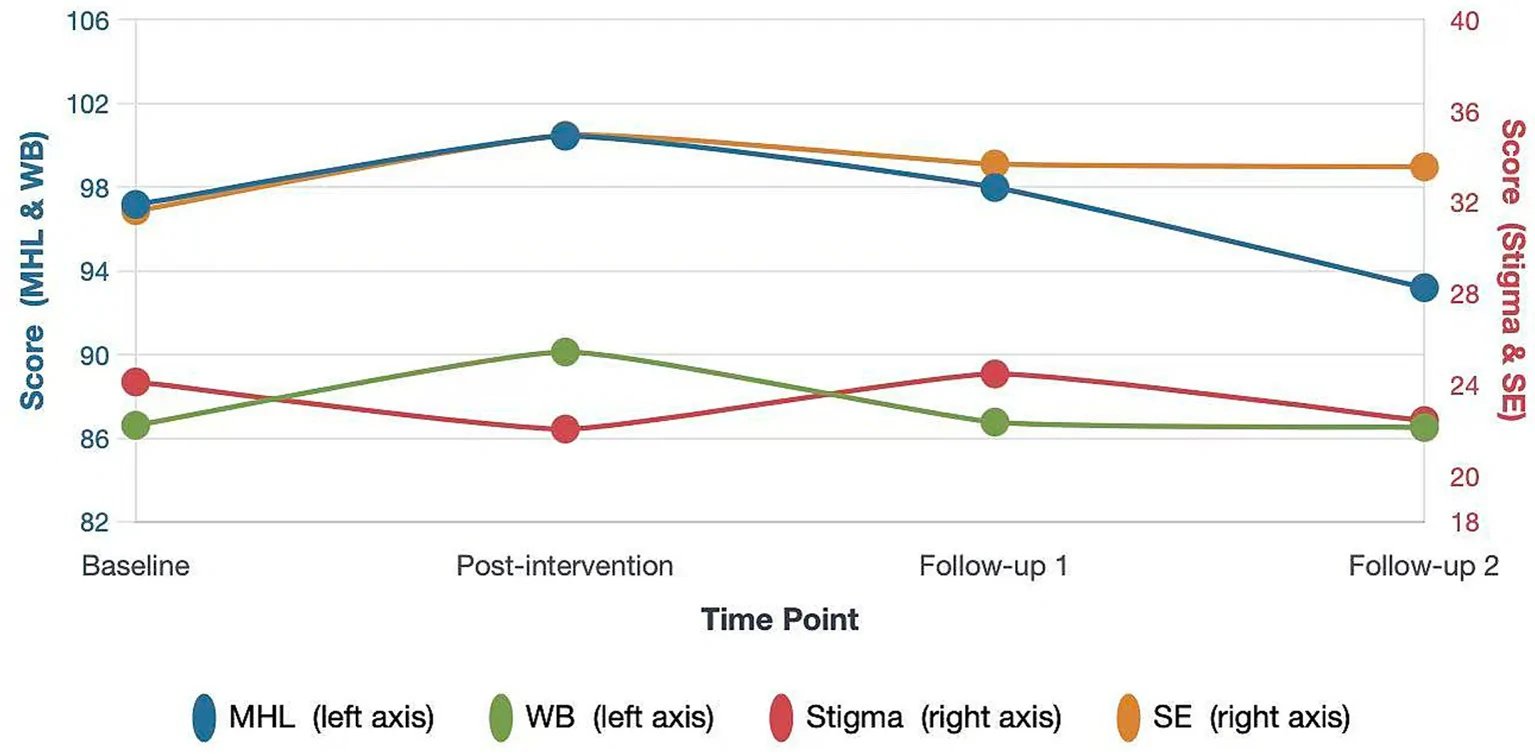
Changes in estimated marginal means of outcome variables over time.

**Figure 3:**
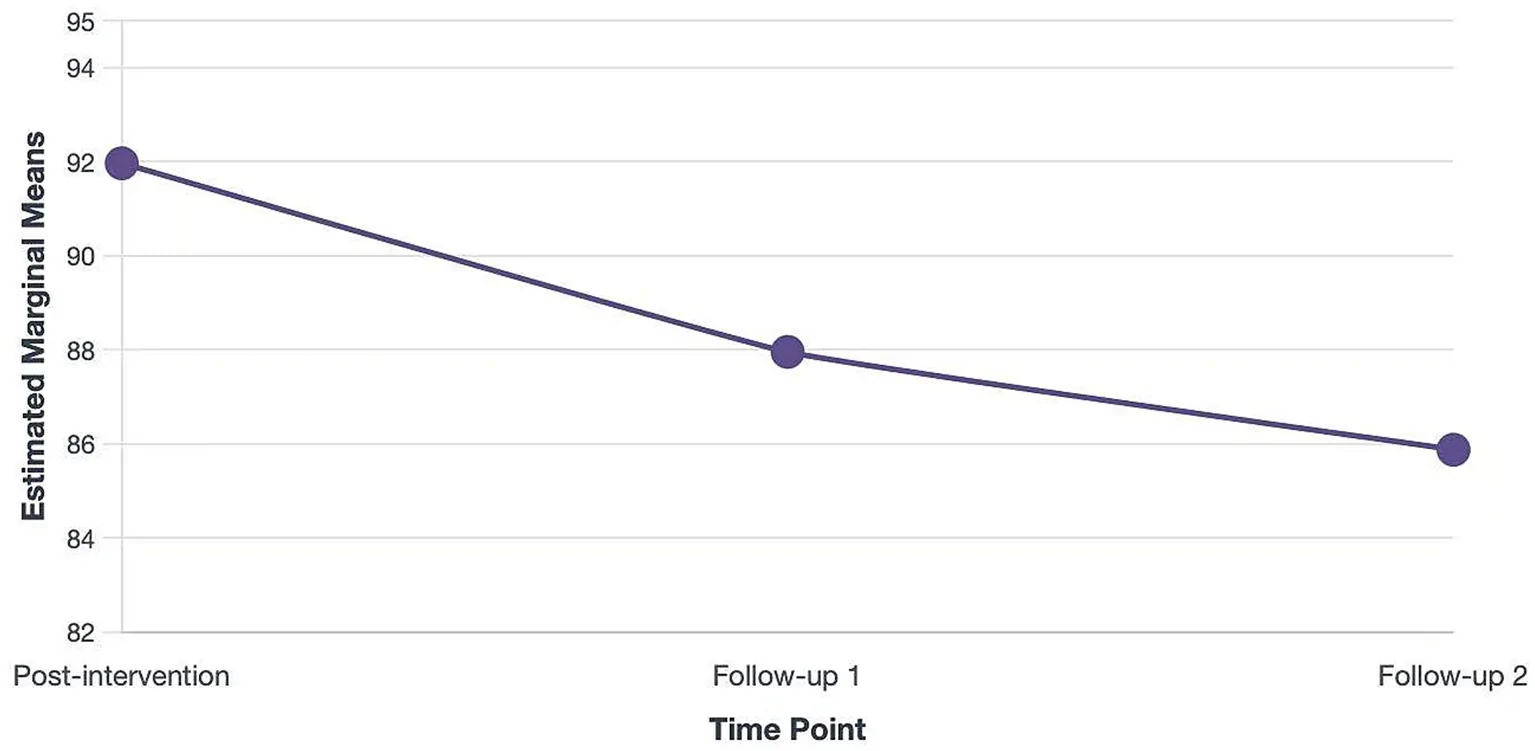
Changes in estimated marginal means of MHFA knowledge scores over time.

The estimated marginal means and standard errors for all outcome variables at the four time points are shown in Table 3. The omnibus fixed effects of time for each outcome variable are summarized in Table 4. Table 5 presents the mean differences, 95% confidence intervals, and Bonferroni-adjusted *p* values for each outcome variable between time points. The longitudinal trends of the outcome variables are depicted in Figure 2, and the trend of MHFA knowledge scores is shown in Figure 3. Overall, self-efficacy significantly improved immediately post-intervention and, although slightly declining during the follow-up period, remained numerically above the baseline level. Mental health literacy increased post-intervention, but this improvement from baseline to post-intervention did not reach statistical significance. The decline from post-intervention to the 3-month follow-up was statistically significant. Self-stigma of seeking help and psychological well-being showed no significant changes across time points. MHFA knowledge exhibited a continuous downward trend from post-intervention through the follow-up period, with the score at the first post-intervention measurement being significantly higher than that at the 3-month follow-up.

**Table 3:** Estimated marginal means (M) ± standard errors (SE) of outcome variables at each time point.

Variable	Baseline T0	Post-intervention T1	Follow-up 1 T2	Follow-up 2 T3
Mental health literacy	97.17 ± 1.60	100.43 ± 1.82	97.98 ± 2.03	93.19 ± 1.87
Self-stigma of seeking help	24.13 ± 1.24	22.07 ± 1.36	24.48 ± 1.49	22.44 ± 1.25
Psychological well-being	86.62 ± 1.59	90.12 ± 1.48	86.77 ± 1.62	86.51 ± 2.34
Self-efficacy	31.63 ± 1.00	34.94 ± 0.73	33.68 ± 0.86	33.55 ± 0.99
MHFA knowledge	—	91.97 ± 0.90	87.96 ± 1.87	85.88 ± 1.92

M, estimated marginal means derived from linear mixed models; SE, standard error. T0, baseline; T1, immediately post-intervention; T2, 1-month follow-up; T3, 3-month follow-up. MHFA knowledge was not assessed at baseline.

**Table 4:** Omnibus fixed effects of time for each outcome variable.

Outcome	Numerator df	Denominator df	*F*	*p*
Mental health literacy	3	18.52	3.75	0.029
Self-stigma of seeking help	3	40.80	2.85	0.049
Psychological well-being	3	38.86	2.32	0.091
Self-efficacy	3	43.79	4.52	0.008
MHFA knowledge	2	44.68	4.37	0.018

Numerator df = number of time points − 1; denominator df estimated using Satterthwaite approximation. All models were fitted using linear mixed models with time as a fixed effect, participant ID as a random intercept, and an unstructured covariance matrix. MHFA knowledge was assessed at three time points only.

**Table 5:** Mean differences (MD), 95% confidence intervals, and adjusted P values for each outcome variable between time points.

Outcome	Baseline → Post-intervention (T0 → T1)	Baseline → 1-month follow-up (T0 → T2)	Baseline → 3-month follow-up (T0 → T3)	Post-intervention → 1-month follow-up (T1 → T2)	Post-intervention → 3-month follow-up (T1 → T3)	1-month → 3-month follow-up (T2 → T3)
**MHL**	3.26 (−1.65, 8.17) *p* = 0.478	0.81 (−5.33, 6.95) *p* = 1.000	−3.98 (−9.01, 1.04) *p* = 0.219	−2.45 (−11.73, 6.84) *p* = 1.000	**−7.24 (−13.07, −1.42)** ***p* = 0.006**	−4.80 (−11.03, 1.43) *p* = 0.242
**Stigma**	−2.06 (−4.84, 0.71) *p* = 0.274	0.34 (−3.72, 4.40) *p* = 1.000	−1.70 (−5.42, 2.03) *p* = 1.000	2.41 (−1.42, 6.24) *p* = 0.534	−0.37 (−4.04, 3.30) *p* = 1.000	−2.04 (−5.05, 0.97) *p* = 0.405
**WB**	3.50 (−0.99, 7.99) *p* = 0.213	0.16 (−4.36, 4.67) *p* = 1.000	−0.11 (−7.07, 6.85) *p* = 1.000	−3.34 (−7.77, 1.09) *p* = 0.249	−3.61 (−9.92, 2.70) *p* = 0.728	−0.27 (−6.25, 5.72) *p* = 1.000
**SE**	**3.31 (0.71, 5.92)** ***p* = 0.006**	2.05 (−0.82, 4.93) *p* = 0.330	1.93 (−1.64, 5.49) *p* = 0.857	−1.26 (−3.72, 1.20) *p* = 0.978	−1.39 (−4.13, 1.36) *p* = 1.000	−0.13 (−3.07, 2.82) *p* = 1.000
**MHFA knowledge** ^**1**^	—	—	—	−4.01 (−8.86, 0.84) *p* = 0.136	**−6.09 (−11.37, −0.81)** ***p* = 0.019**	−2.08 (−7.19, 3.03) *p* = 0.952

MD = estimated marginal mean difference (later minus earlier; positive = increase). Data in parentheses are 95% CI. *p*-values were Bonferroni-corrected; bold indicates *p* < 0.05. MHL, mental health literacy; WB, psychological well-being; SE, self-efficacy; T0, baseline; T1, post-intervention; T2, 1-month follow-up; T3, 3-month follow-up. ^1^MHFA knowledge assessed only at T1, T2, and T3.

### 3.4 Sensitivity analysis

To assess the robustness of the main findings, a sensitivity analysis was conducted by additionally including baseline self-stigma of seeking help (Stigma_T0) as a fixed covariate in the linear mixed models. After adjustment, the post-intervention to 3-month follow-up comparison for MHL remained statistically significant (*p* = 0.034), and the baseline to post-intervention comparison for self-efficacy also remained significant (*p* = 0.009). All pairwise comparisons for psychological well-being remained non-significant. The covariate Stigma_T0 was not statistically significant in any of the three models (*p* values ranging from 0.212 to 0.709).

Second, a complete-case analysis including only the 35 participants who completed all four assessments was conducted. For mental health literacy, self-stigma of seeking help, and psychological well-being, the complete-case analysis yielded results consistent with those of the main analysis. For self-efficacy, the main analysis showed a significant overall time effect (*p* = 0.017), whereas the complete-case analysis yielded a *p*-value of 0.056, which was slightly above the conventional threshold of 0.05. The direction of change for self-efficacy in the complete-case analysis was consistent with that of the main analysis. The reduced statistical significance is likely attributable to the smaller sample size, which decreases statistical power.

Taken together, the sensitivity analyses support the robustness of the main findings.

## 4 Discussion

This study found that a 3-day face-to-face MHFA training program was associated with significant improvements in nursing students’ self-efficacy immediately post-intervention, though these improvements did not persist significantly over the 3-month follow-up. Mental health literacy increased after the intervention but this improvement did not reach statistical significance, and a significant decline was observed from post-intervention to the 3-month follow-up. MHFA knowledge scores showed a continuous downward trend during the follow-up period. No significant changes were observed in self-stigma of seeking help or psychological well-being.

This study found that participants’ self-efficacy significantly improved from baseline to immediately post-intervention, which is consistent with findings from previous studies on psychological first aid and MHFA training. Bayageldi and Binici (16) reported that nursing students’ general self-efficacy increased significantly from 20.51 to 23.24 after psychological first aid training, a finding consistent with McGregor et al. (17), who found that MHFA training significantly enhanced nursing students’ confidence in providing clinical assistance. This improvement may be attributed to the interactive teaching methods widely adopted in the training program. Bayageldi and Binici (16) employed a combination of lectures, video demonstrations, situational teaching, and case-based learning and first demonstrated the positive effects of integrating case-based learning with simulation exercises on nursing students. Azizi et al. (18) confirmed through a quasi-experimental design that simulation-based teaching could explain 58% of the variance in self-efficacy, with its mechanism lying in providing learners with opportunities to practice repeatedly and accumulate successful experiences in a safe environment. This aligns with the core proposition of Bandura’s self-efficacy theory (19) that mastery experience is the most important source of self-efficacy. The ALGEE simulation exercises in the present study provided such mastery experiences. Although the self-efficacy scale used in this study was derived from the 5A Mental Health Ambassador Program, its items assess confidence in performing core supportive behaviors, such as identifying mental health needs, encouraging professional help-seeking, and following up on emotional states, which correspond closely to the key steps of the ALGEE action plan taught in the training. Previous MHFA research has similarly operationalized self-efficacy using items directly aligned with ALGEE behaviors, suggesting the potential appropriateness of this approach (20). The high internal consistency of the scale in the present study (Cronbach’s *α* = 0.941) indicates satisfactory reliability for the study sample. However, self-efficacy showed a slight decline at the 3-month follow-up in the present study, and the differences from baseline at the follow-up time points were not statistically significant. This pattern suggests that a short-term intensive training program has limited capacity to sustain the initial improvements over time. Azizi et al. (18) also noted that their study evaluated only short-term effects without long-term follow-up and emphasized that competence development requires sustained practice and accumulation. Future research should consider incorporating regular booster sessions or blended reinforcement models combining online and offline formats, and extending the follow-up duration to identify effective strategies for sustaining the long-term effects of self-efficacy improvement.

This study found that mental health literacy increased following the intervention, but the improvement did not reach statistical significance, a finding inconsistent with previous MHL intervention studies. Kurki et al. (21) reported that the digital Transitions program for first-year medical students significantly improved mental health knowledge from baseline to both post-intervention and the 2-month follow-up, with similar effects observed in the original Canadian version. Reavley et al. (22) further confirmed in a randomized controlled trial of MHFA training in the workplace that blended delivery was superior to fully online training in terms of knowledge retention and helping intentions. This discrepancy may first be attributable to differences in training duration and delivery format. Although the Transitions program in Kurki et al. (21) included only two 60-min face-to-face lectures, students had access to online self-learning materials for 4 weeks between lectures, allowing for repeated review. In contrast, the training in the present study was delivered over three consecutive days, lacking distributed learning opportunities and a time window for reinforcement. The blended MHFA training in Reavley et al. (22) consisted of a 6-h online course combined with 4 h of face-to-face reinforcement, with a total duration substantially longer than that of the present study. Furthermore, the baseline MHL score of participants in this study was 97.17 (out of a maximum of 132), which was already at a relatively high level. Based on the MHLS-C norms established by Wang et al. (12) in a sample of 872 clinical nurses, this score approached the norm median of 99, suggesting that participants already possessed a level of mental health literacy comparable to the moderate level of practicing nurses prior to the training. In addition, 57.14% of participants in this study had previously completed mental health-related courses, primarily Psychiatric Nursing, and prior research has identified coursework in psychology or psychiatry as a significant positive predictor of mental health literacy among nursing students (23). Therefore, the non-significant improvement in MHL observed in this study may not reflect a lack of intervention effectiveness, but rather the limited capacity of a short-term generic training program to produce substantial gains beyond an already relatively high baseline. This interpretation is consistent with the findings reported by Kurki et al. (21) and Selim et al. (24), in which intervention effects were similarly constrained in samples with high baseline scores. In this study, MHL scores declined significantly from post-intervention to the 3-month follow-up, returning to a level that did not differ significantly from baseline. This pattern aligns with the findings of Gine-Cipres et al. (25), who reported that knowledge scores after mindfulness training returned to baseline levels at the 3-month follow-up, underscoring the challenge of sustaining knowledge gains after short-term intensive training without reinforcement. Future research should therefore optimize training duration and include samples with varying baseline MHL levels to more comprehensively evaluate the effectiveness and durability of MHL interventions.

This study found that self-stigma of seeking help did not change significantly across time points, which is consistent with the results reported by Kurki et al. (21), whose digital Transitions program also observed no change in stigma. A meta-analysis of MHFA training effectiveness by Morgan et al. (7) similarly showed that the training produced only small effect sizes for stigma reduction, which diminished to non-significance at the 12-month follow-up, suggesting that MHFA training has limited and unstable effects on reducing stigma. Regarding training content, Morgan et al. (7) noted that the MHFA program primarily focuses on teaching participants to recognize symptoms of mental disorders, provide initial help, and guide individuals toward professional support. Because the MHFA curriculum is oriented toward helping others, whereas the SSOSH measures internalized attitudes toward seeking personal psychological support, the training may have limited capacity to change this specific type of stigma. Cultural factors may also play a role in these findings. A survey of Chinese university students showed that mental illness is often heavily stigmatized in Chinese society due to Confucian values such as collectivism and concern for family reputation, resulting in higher levels of stigma compared with Western countries (26). Short-term generic training programs may be insufficient to substantially alter deeply ingrained stigmatizing attitudes within a cultural context. Future research could draw on the culturally tailored anti-stigma intervention for Chinese nursing students developed by Chen et al. (27) and incorporate local cultural elements into the MHFA training design. As noted in the scoping review by Zhang et al. (28), the methodological quality of interventional research in this field still requires improvement, and developing standardized assessment tools and exploring intervention strategies beyond psychoeducation represent important directions for future work.

The present study found that psychological well-being did not change significantly across any of the time points, with only a transient upward trend immediately after the intervention before returning to baseline levels. This finding may be related to the trait-like nature of psychological well-being. Kubzansky et al. (29) noted that most studies in this area have tracked outcomes for <6 months, with none exceeding 12 months, and that evidence on the durability of intervention effects remains insufficient. Wang and Mei (30) similarly reported that a positive psychological intervention for nursing students produced only short-term effects on perceived stress and optimism, with no significant improvements observed in more stable positive psychological traits such as hope, a pattern consistent with the trajectories of psychological well-being observed in the present study. Regarding measurement tools, the present study used the 18-item short form of Ryff’s Scales of Psychological Well-being. A meta-analysis by van Dierendonck and Lam (31) demonstrated that interventions based on Ryff’s model can enhance eudaimonic well-being, yet the strongest effects were observed for integral programs directly linked to Ryff’s conceptual model rather than short-term generic training using a single technique. The meta-analysis also found that different versions of Ryff’s scales yielded varying effect sizes, with the 18-item version producing a smaller total effect size than the 42-item version. Therefore, the absence of significant changes in psychological well-being in this study may reflect the limited capacity of short-term generic training to alter long-term well-being traits rather than a lack of intervention effectiveness perse.

MHFA knowledge scores in this study showed a continuous downward trend from post-intervention through the follow-up period, with the first post-intervention score being significantly higher than that at the 3-month follow-up, indicating a modest but statistically significant decline in knowledge scores during the follow-up period. The magnitude of this decline, approximately 6 points on a 100-point scale, suggests that its clinical relevance may be limited. Häger et al. (32) similarly found that knowledge scores among Nordic healthcare professionals declined significantly from post-intervention to the 6-month follow-up after MHFA training, with training effects diminishing over time and requiring regular repetition to be maintained. Bharne et al. (33) demonstrated that brief booster videos can effectively reverse this decline, suggesting that periodic review sessions may be a feasible strategy for mitigating knowledge decay. Future research should incorporate standardized assessment tools at baseline and explore key factors influencing long-term knowledge retention. This study had two limitations in the assessment of MHFA knowledge. The knowledge test was not administered at baseline, thus precluding evaluation of immediate knowledge gains from the intervention, and the potential influence of confounding effects such as regression to the mean should also be considered. In addition, the test was a self-designed instrument developed by the research team, and its reliability and validity have yet to be established, which may affect the accuracy of measurement compared with standardized tools. The validation study of an MHFA knowledge questionnaire for Chinese non-psychiatric nurses by Li et al. (34) underscores the importance of using standardized, psychometrically validated instruments. Furthermore, the training was delivered in English using the Hong Kong version of the MHFA curriculum, with research team members providing on-site translation. Although this arrangement was intended to facilitate comprehension, varying levels of English proficiency among participants may have affected their understanding of the training content and their subsequent performance on the knowledge test. This language-related confound represents a methodological limitation that should be considered when interpreting the observed decline in knowledge scores. Future research using a localized, Chinese-language version of the MHFA curriculum would help clarify the extent to which language barriers versus actual knowledge retention contribute to the observed patterns.

## 5 Strengths and limitations

The main strengths of this study include the inclusion of four assessment time points with a 3-month follow-up, which allows for a more comprehensive capture of the dynamic changes in intervention effects; the use of linear mixed models with restricted maximum likelihood estimation to handle missing data, effectively utilizing all available participant information and reducing selection bias; and the implementation of the intervention in an authentic nursing education setting, delivered by the same team of certified instructors using standardized training materials, which ensured intervention consistency and lends the findings practical relevance for nursing education.

The most notable limitation of this study is the absence of a control group, which precludes definitive conclusions regarding the true effectiveness of the intervention. Second, the sample size was limited and drawn from a single institution, and the sample was predominantly female. The generalizability of the findings to other nursing schools, regions, or to male nursing students therefore warrants caution. The training was delivered in English using the Hong Kong version of the MHFA curriculum, and language and cultural differences may have affected participants’ comprehension and assimilation of certain content. Another major limitation concerns the MHFA knowledge test, which was developed by the research team and has not been systematically validated. Formal psychometric evaluation, including pilot testing and expert review, was not conducted prior to its use in this study. Moreover, it was not administered at baseline, preventing evaluation of immediate knowledge gains from the intervention. The use of standardized, validated instruments is recommended for future research to strengthen the measurement of MHFA knowledge. Finally, the follow-up period was limited to 3 months, which precludes evaluation of the longer-term sustainability of the training effects.

Future research should adopt a randomized controlled design with larger samples drawn from multiple institutions and extend the follow-up period to assess long-term sustainability. Subsequent studies could also incorporate qualitative interviews with trainees to gain deeper insight into specific barriers to curriculum comprehension, thereby providing a foundation for the development of a localized MHFA training program tailored to the Chinese context.

## 6 Implications

This study found that short-term MHFA training effectively enhanced nursing students’ self-efficacy, suggesting that such training could serve as a supplementary module within nursing curricula to help students build professional confidence in providing mental health support to others. However, the continuous decline in MHFA knowledge scores during the follow-up period reflects the limitations of short-term intensive training in sustaining knowledge retention. Future curriculum design could incorporate regular review sessions or online reinforcement resources to mitigate knowledge decay. In addition, the training was delivered in English using the Hong Kong version of the MHFA curriculum, and language and cultural differences may have affected students’ comprehension and assimilation. A localized MHFA training program tailored to the language habits and cultural context of mainland Chinese nursing students should be developed in future research.

With the advancement of digital technology, online mental health interventions have demonstrated unique advantages. Future research could explore a blended online and offline MHFA training model, using digital tools to support knowledge consolidation and continuous reinforcement during the follow-up period, thereby expanding both the reach and sustainability of the training. As the participants in this study were predominantly postgraduate nursing students with medical backgrounds whose baseline mental health literacy was already at a moderately high level, future research could also consider extending MHFA training to non-medical student populations to explore its applicability and effectiveness in broader groups.

## 7 Conclusion

This study showed that a 3-day face-to-face MHFA training program was followed by a significant increase in nursing students’ self-efficacy immediately post-intervention, though this improvement did not persist significantly over the 3-month follow-up. The training showed insufficient effects on mental health literacy, self-stigma of seeking help, and psychological well-being, and a notable decay in MHFA knowledge was observed during the follow-up period. Future efforts should focus on developing a localized MHFA training program and integrating it as a supplementary module within nursing curricula to strengthen students’ confidence in providing mental health support to others.

## Data Availability

The original contributions presented in the study are included in the article/supplementary material, further inquiries can be directed to the corresponding authors.
