# Correction: Effectiveness of mental health first aid training for nursing students in northwestern China: a quasi-experimental study

**DOI:** 10.3389/fmed.2026.1982045

**Published:** 2026-09-10

**Authors:** Shadiyemu Tuerxun, Xi Chen, Mingzhe Wu, Haijing Ma, Yujia Chen, Ping Song, Yunxia Li, Yim-Wah Mak

**Affiliations:** 1 School of Nursing, Lanzhou University, Lanzhou, China; 2 School of Nursing, The Hong Kong Polytechnic University, Kowloon, Hong Kong SAR, China; 3 Department of Interventional Medicine, The First Hospital of Lanzhou University, Lanzhou, China

**Keywords:** mental health first aid (MHFA), mental health literacy, nursing students, quasi-experimental study, self-efficacy

The funder ¨2025 Lanzhou University “PIONEERING DISCIPLINE PARTNERSHIP PROJECT”, No. 561119501¨ was erroneously included.

The **corrected funding** statement reads:

¨The author(s) declared that financial support was received for this work and/or its publication. This study was supported by the Youth Science and Technology Fund of the Natural Science Foundation of Gansu Province (No. 26JRRA231).¨

The original version of this article has been updated.

